# Importance of Welfare and Ethics Competence Regarding Animals Kept for Scientific Purposes to Veterinary Students in Australia and New Zealand

**DOI:** 10.3390/vetsci5030066

**Published:** 2018-07-14

**Authors:** Teresa Collins, Amelia Cornish, Jennifer Hood, Chris Degeling, Andrew D. Fisher, Rafael Freire, Susan J. Hazel, Jane Johnson, Janice K. F. Lloyd, Clive J. Phillips, Vicky Tzioumis, Paul D. McGreevy

**Affiliations:** 1 School of Veterinary and Life Science, Murdoch University, Murdoch, WA 6150, Australia; jenih@iinet.net.au; 2 Sydney School of Veterinary Science, University of Sydney, Sydney, NSW 2006, Australia; acor3786@uni.sydney.edu.au (A.C.); vicky.tzioumis@sydney.edu.au (V.T.); paul.mcgreevy@sydney.edu.au (P.D.M.); 3 Research for Social Change, Faculty of Social Science, University of Wollongong, Wollongong, NSW 2522, Australia; degeling@uow.edu.au; 4 Faculty of Veterinary and Agricultural Sciences, University of Melbourne, Parkville, VIC 3010, Australia; adfisher@unimelb.edu.au; 5 School of Animal and Veterinary Science, Charles Sturt University, Wagga Wagga, NSW 2650, Australia; rfreire@csu.edu.au; 6 School of Animal and Veterinary Science, University of Adelaide, Roseworthy, SA 5005, Australia; susan.hazel@adelaide.edu.au; 7 Department of Philosophy, Macquarie University, Sydney, NSW 2109, Australia; jane.johnson@mq.edu.au; 8 College of Public Health, Medical and Veterinary Science, James Cook University, Townsville, QLD 4811, Australia; janice.lloyd@jcu.edu.au; 9 School of Veterinary Science, University of Queensland, Gatton, QLD 4343, Australia; c.phillips@uq.edu.au

**Keywords:** animal welfare, veterinary education, Day One Competence, gender, veterinary ethics, humane endpoint, 3Rs, research animals, euthanasia, conscientious objection

## Abstract

Veterinarians are in a strong position of social influence on animal-related issues. Hence, veterinary schools have an opportunity to raise animal health and welfare standards by improving veterinary students’ animal welfare and ethics (AWE) education, including that related to animals used for scientific purposes. A survey of 818 students in the early, mid, and senior stages of their courses at all eight veterinary schools across Australia and New Zealand was undertaken on their first day of practice (or Day One Competences) to explore how veterinary students viewed the importance of their competence in the management of welfare and ethical decision-making relating to animals kept for scientific purposes. From highest to lowest, the rankings they assigned were: *Animal Ethics Committee (AEC) Procedures or Requirements*; *3Rs (Replacement, Refinement and Reduction)*; *Humane Endpoints*; *Euthanasia*; *“What Is a Research Animal?”*; and *Conscientious Objections*. Female students rated *Conscientious Objections*, *Humane Endpoints*, and *Euthanasia* significantly higher than male students did across the three stages of study. The score patterns for these three variates showed a trend for the male students to be more likely to score these topics as extremely important as they advanced through the course, but female students’ scores tended to decline slightly or stay relatively stable. No gender differences emerged for the three variates: *3Rs (Replacement, Refinement and Reduction)*; *AEC Procedures or Requirements*; and “*What Is a Research Animal?*”. This study demonstrates that understandings of the regulatory and normative frameworks are considered most important in animal welfare and ethics competence in veterinary students. To the authors’ knowledge, this is the first study to investigate what importance veterinary students place on their competence regarding animals kept for scientific purposes.

## 1. Introduction

Animal welfare and ethics (AWE) is an integral and growing component of modern veterinary education [1]. The reasons for this include an increasing community concern about animal welfare [2,3] and growing public expectation that veterinarians demonstrate competency and knowledge in AWE principles underpinning the societal use of animals [4], including the use of animals for scientific purposes. Given veterinarians attain the skills and knowledge required to provide animal care and disease management for a wide range of species, they are ideally placed to: (i) understand the potential impacts of many scientific procedures on animals; and (ii) ensure the welfare of the animals affected. Veterinarians can provide a valued and unique perspective on numerous areas of animal use, including the challenging issues that arise when animals are used for scientific purposes.

Although the use of animals for scientific purposes still attracts debate, as described below, the current regulatory position in most western countries allows regulated animal use to occur because of the perceived benefits in generating new knowledge and in the development of therapies for humans and animals alike [5]. Animals kept for scientific purposes include those used for the education of animal science, biomedical, and veterinary students in a variety of contexts. In Australia, and around the world, animals used for research or teaching are not limited to laboratory rodents; many species are used, including livestock, birds, cats, dogs, poultry, reptiles, fish, and wildlife [6].

The use of animals for scientific purposes engenders a wide range of ethical perspectives, with some people seeking the complete abolition of animal use, and others strongly supporting their continued use (reviewed by Ormandy and Schuppli) [7]. Although regulatory systems vary from country to country, in most jurisdictions, research and teaching institutions are required to ensure that staff and students using animals for scientific purposes are appropriately trained, that animals are well cared for, and that the ethical review process for projects is robust. Hence, this is a growing area of involvement for veterinarians, including their roles as: educators (to public, clients, new researchers, and industry); researchers or teaching academics; members of institutional animal ethics committees or their equivalents; technical consultants for research teams or animal facility managers; health and welfare consultants in regional and international areas; government veterinarians; welfare organization veterinarians; and regulators.

Increasingly, the public is looking to veterinarians, given their advanced scientific knowledge and experience [8], to lead as animal welfare advocates responsible for safeguarding the health and welfare of all animals [9,10]. Animal welfare captures all of the conditions that may affect the physical and emotional state of the animal, its ability to cope, and its quality of life [11]. Welfare embraces the ethics of animal use, the science of animal behavior, and the regulatory framework within which the utilization of animals occurs. Further, veterinarians are in a strong position of leadership and social influence on animal-related issues, and veterinary schools have an opportunity to promote informed welfare advocacy and related policy-making by improving veterinary students’ education in AWE [10,12,13].

The world is facing challenges from climate change, habitat destruction, threats to food availability and security, and public health risks—all of which highlight the need for veterinarians to work within the One Health and One Welfare frameworks to achieve sustainable and holistic health and welfare improvements for animals, people, and the environment [14,15]. As society seeks to find solutions to rising global challenges, there is also growing recognition of the need for medicine, surgery, and the use of animals in research to be conducted with guidance from current evidence-based welfare science and normative animal ethics frameworks. Genetically modified animals now make up a significant proportion of animals kept for scientific purposes, which provides new and more complex challenges for ethical decision-making [8,16]. In addition, a practical understanding of AWE is increasingly likely to be mandated through accreditation of veterinary teaching programs, with the UK’s Royal College of Veterinary Surgeons (RCVS) Day One Competences already requiring skills related to animal welfare [17].

Research suggests that veterinary students’ empathy for animals declines during the years spent at university, but the reasons for this are not well understood. One Australian study by Pollard-Williams et al. [18] showed that empathy for animals declined between first and fifth year and was lower in male than in female students. Moreover, Phillips and McCulloch [19] found that female students showed greater concern for the reverence of animal life than males did. Understanding the topics of most concern to current students and the influence of trends in student demographics allows educators to prepare activities that promote discussion based on realistic and perceived relevant AWE issues.

While it is clear that the use of animals for scientific purposes is an important area, there is little published information on the attitudes of veterinary students to it or on their understanding of its importance to them as veterinarians. To address this and to better prepare veterinarians to meet societal needs, a survey of AWE attitudes of veterinary students in Australia and New Zealand was undertaken. The aims were to (i) understand what veterinary students consider important topics for Day One Competences when dealing with animals kept for scientific purposes; and (ii) ascertain how students’ stated priorities might be affected by gender and stage of study.

## 2. Materials and Methods

### 2.1. Study Participants

Students enrolled in veterinary science or veterinary medicine courses at all universities in Australia and New Zealand during October 2014 (*n* = 3320) were invited to participate in the survey. Institutional human ethics approval was granted by the University of Sydney Human Ethics Committee and by participating universities prior to the start of data gathering (approval number: 2014/739).

### 2.2. The Questionnaire

At a 2-day face-to-face workshop, faculty members teaching AWE at each of the veterinary schools in Australia and New Zealand formulated a list of key topics relevant to the use of animals kept for scientific purposes (in addition to other categories of animal use). These topics formed a component of the broader questionnaire that asked students to rank the importance of topics across a range of themes. The survey system SurveyMonkey (www.surveymonkey.com) was used to administer the online questionnaire for a period of approximately 1 month, from 9 October to 14 November 2014. The voluntary participation of students was sought via a series of three emails that included a link to the questionnaire. Following the initial email, a reminder was sent to students 1 and 2 weeks later. The link to the survey was closed 1 week after the final reminder. An award of AUD $200 to the representative student body at the institution with the highest participation rate provided an incentive for students to complete the survey. 

The questionnaire comprised 12 multiple-choice questions covering several types of animal use. The first four questions related to consent to participate and student demographics (e.g., university, gender, and year of study). The survey then defined the RCVS Day One Competences, listing the skills and knowledge students should expect to have on the first day of practice. Questions 5–10 covered other topics, not reported in this paper, with Question 11 focusing on the category of animals kept for scientific purposes. Using a 10-point Likert scale, from *extremely important* (1) to *least important* (10), students were asked to indicate how important an understanding of each AWE topic for animals kept for scientific purposes was for veterinarians on their first day of practice. 

AWE topics important for Day One Competences were identified at the face-to-face workshop, and the topics that students were asked to prioritize in the survey were: the *3Rs (Replacement, Refinement and Reduction)*; *AEC (Animal Ethics Committee) Procedures or Requirements*; *Conscientious Objections*; *Humane Endpoints*; *“What Is a Research Animal?”* and *Euthanasia*. An explanation for these topics is provided below (but, to avoid leading the students in any way, it was not included in the questionnaire itself).

### 2.3. 3Rs (Replacement, Refinement and Reduction)

The principles of Replacement, Refinement and Reduction—known as the 3Rs—were first proposed by Russell and Burch in 1959 [20] and now have almost universal acceptance. The basic tenet of the 3Rs is that the humane treatment of experimental animals is actually a prerequisite for valid research outcomes. Replacement involves seeking non-sentient alternatives to avoid or replace sentient animals in research. Refinement has been defined as those methods which avoid or minimize the pain or suffering of the animals involved, while reduction involves minimizing the number of animals used per experiment or study (https://www.nc3rs.org.uk/the-3rs).

### 2.4. AEC (Animal Ethics Committee) Procedures or Requirements

Before any use of animals for scientific purposes can take place in Australia or New Zealand, approval must be obtained from a properly constituted AEC. The AEC’s main role is to review and approve or reject animal use proposals. Like institutions and their staff and students, AECs are also legally bound by the Australian Code for the Care and Use of Animals for Scientific Purposes (8th Ed. 2013) and Part 6 of the New Zealand Animal Welfare Act 1999, which govern the use of animals for scientific purposes in Australia and New Zealand respectively.

### 2.5. Conscientious Objections

A conscientious objection to the use of animals for scientific purposes involves an individual refusing to participate in scientific activities involving animals on the basis of ethical or religious grounds. Allowing students to conscientiously object to undertaking practices that use, harm, or kill animals on the grounds of ethical or religious beliefs are increasingly common in education institutes, and can be viewed as essential to students’ enjoyment of and participation in science in a way that does not conflict with their ethics or religion.

### 2.6. Humane Endpoints

Humane endpoints are the earliest indicators in an animal experiment of (potential) pain and/or distress that, within the context of moral justification, can be used to avoid or limit pain and/or distress by taking actions such as humane killing or terminating, or alleviating the pain and distress [21]. Applying humane endpoints must be considered when animal experiments involve severe pain and suffering.

### 2.7. “What Is a Research Animal?”

A research animal is any animal that is used for research purposes, including non-human primates, other mammals, birds, wildlife, reptiles, and invertebrates [22]. Research includes all activities conducted with the aim of acquiring, developing, or demonstrating knowledge or techniques in all areas of science [22]. 

### 2.8. Euthanasia

Euthanasia, or humane killing, is the act of inducing death using a method appropriate to the species that results in a rapid loss of consciousness without recovery with minimum pain and/or distress to the animal [22]. In research projects, the need for euthanasia of an animal on humane grounds may arise from unforeseen circumstances or may be a planned endpoint.

### 2.9. Data Management

Given some difference in course structure across the universities, the responses to Question 4, which asked students to identify their year of study, were recoded with Years 1 and 2 as early students, Years 3 and 4 as mid students, and Years 5 and 6 as senior students.

For each question, there were two factors—gender and stage of study—which resulted in six combinations of students, each indicating a level of importance to a question on a 1–10 scale, with 1 representing *extremely important* and 10 being *least important*.

### 2.10. Statistical Analysis

All data were checked for errors, and the cleaned data were entered into GenStat Version 15 (VSN International, Hemel Hempstead, UK). A statistical analysis was performed to test differences in responses associated with (i) gender; and (ii) stage of study. Interest lay in whether males and females (gender) at three levels of study (early, mid, senior) differed in their distribution of scores. 

Ordinal regression failed to effectively model responses to these variates, so we used log-linear modeling to analyze the three-way contingency table of frequencies. When the three-factor interaction was not significant, the P values for the two-factor interactions were the same as those for the maximum likelihood chi-squared test in a two-way contingency table. However, log-linear modeling relies on expected frequencies that are not too small, generally not less than 1. Given that there were only 13 male student respondents in the senior years of study across all universities, this needed to be addressed. To manage this, scores of 6 or more were combined for the analysis, since there were very few students who ranked variates in this way. However, the plots in this report are based on the percentages of all scores.

## 3. Results

### 3.1. Student Demographics

Of the 3320 students emailed, 818 participated in the voluntary survey, providing an overall average response rate of 25%. There were 673 (82%) females and 145 (18%) males. The number of respondents is shown in Table 1.

### 3.2. Survey Responses

Question 11 of the survey asked students: How important is an understanding of the following topics for veterinarians on their first day in practice with Animals Kept for Scientific Purposes? 

The overall rankings of the six AWE topics are listed in Table 2. For each item in the questionnaire, the average ranking was derived by multiplying each rank (1–10) by the number of students that chose it, summing these scores for each item and dividing by the total number of students (*n*) that responded to that item. Students ranked *AEC Procedures or Requirements* as the most important topic, followed by the *3Rs*, and then *Humane Endpoints*. These three topics were ranked consistently as highly important across all the stages of study (range 2.21–2.43). The least important topics, in ranked order, were *Euthanasia*, *“What Is a Research Animal?”* and *Conscientious Objections*.

### 3.3. Analysis of Gender and Stage of Study

**Figure 1:**
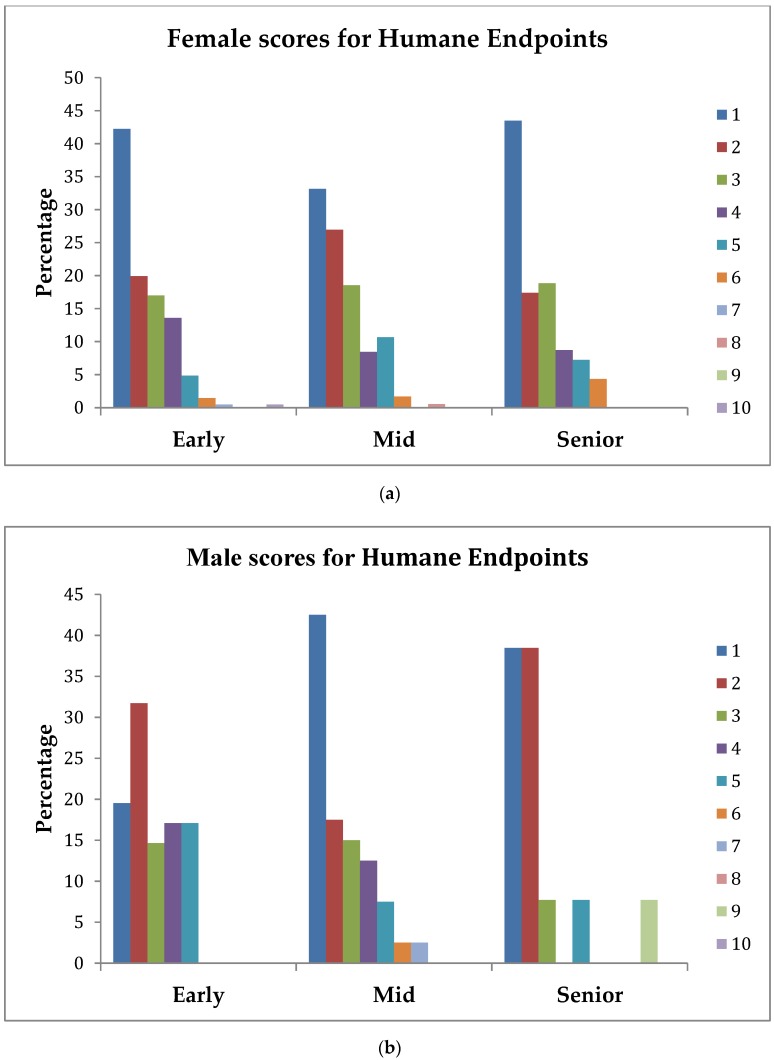
(**a**) Percentage of female students across stage-of-study rating the importance (1 = extremely important, 10 = least important) of an understanding of Humane Endpoints on their first day in practice; (**b**) percentage of male students across stage-of-study rating the importance (1 = extremely important, 10 = least important) of an understanding of Humane Endpoints on their first day in practice.

**Figure 2:**
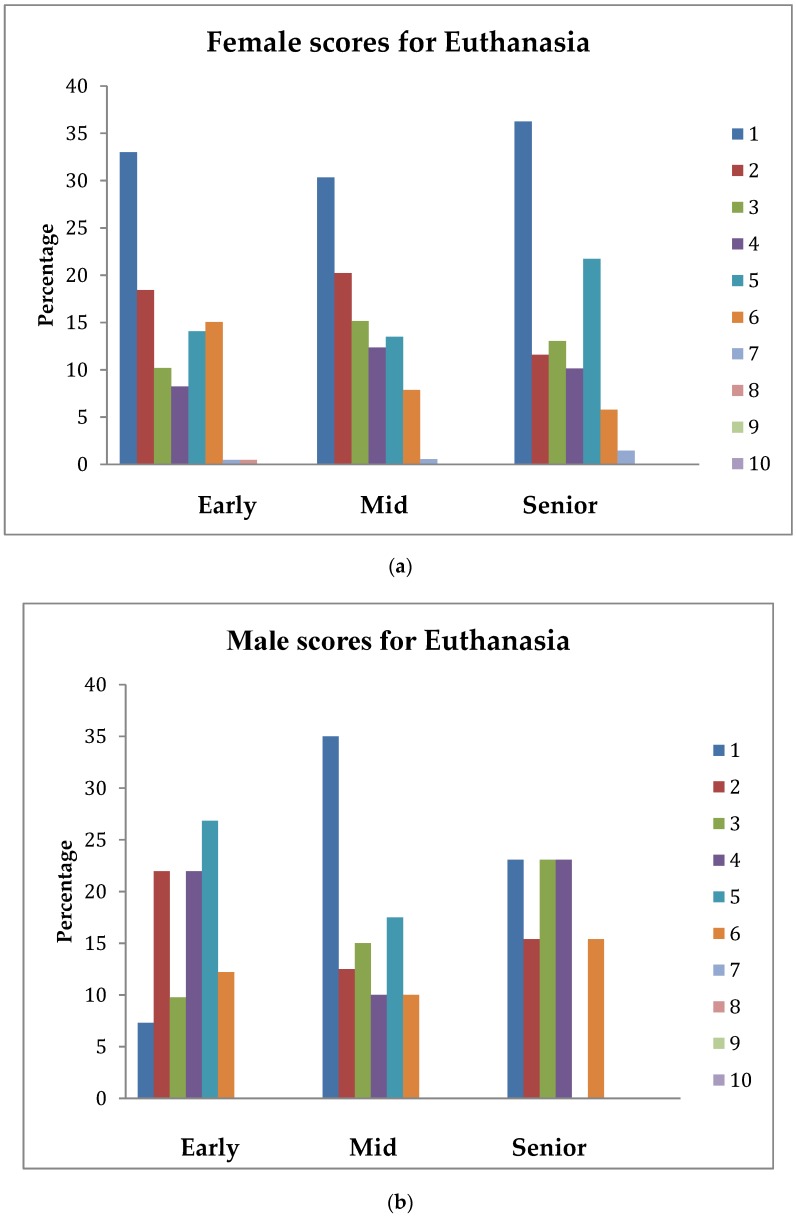
(**a**) Percentage of female students across stage-of-study rating the importance (1 = extremely important, 10 = least important) of an understanding of Euthanasia on their first day in practice; (**b**) percentage of male students across stage-of-study rating the importance (1 = extremely important, 10 = least important) of an understanding of Euthanasia on their first day in practice.

**Figure 3:**
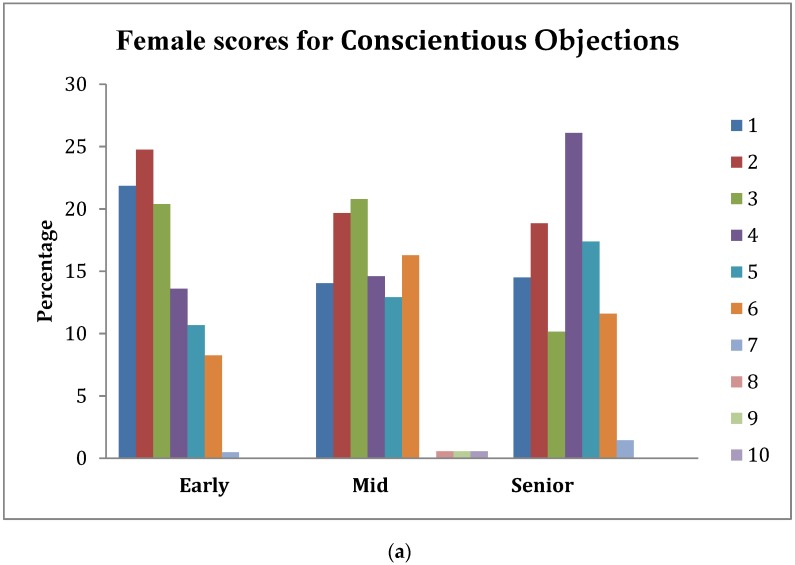

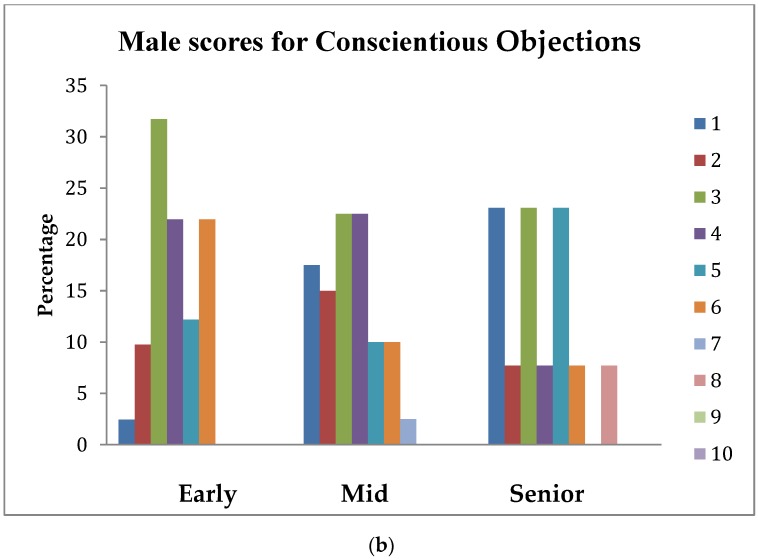
(**a**) Percentage of female students across stage-of-study rating the importance (1 = extremely important, 10 = least important) of an understanding of Conscientious Objections on their first day in practice; (**b**) percentage of male students across stage-of-study rating the importance (1 = extremely important, 10 = least important) of an understanding of Conscientious Objections on their first day in practice.

Analysis of the topics *Humane Endpoints*, *Euthanasia*, and *Conscientious Objection* showed there was significant interaction between gender and stage of study (as shown in Table 2). Hence, the patterns of percentage scores across the study for these variates for females and males were examined separately and are shown in Figure 1, Figure 2 and Figure 3. 

### 3.4. Humane Endpoints

The interaction between gender and stage of study was significant (*p* = 0.026). For female early, mid, and senior students, 62%, 60%, and 61%, respectively, ranked this topic as being *extremely important* (score 1) or only slightly less so (score 2). For male early, mid, and senior students, this topic rose in importance across the stages of study, as it was ranked 1 or 2 by 51%, 60%, and 77%, respectively. Female students at all stages ranked this topic as 6 or lower on the 1–10 scale, whereas male early, mid, and senior students ranked this as 5 or lower, 7 or lower, or 9 or lower, respectively. The greatest difference in the scoring patterns was between male and female early students, with approximately 42% of female but only 20% of male students prioritizing this topic as *extremely important* (1).

### 3.5. Euthanasia

The interaction between gender and stage of study was significant (*p* = 0.014). The data show that approximately 51%, 55%, and 48% of female early, mid, and senior students, respectively, scored an understanding of this topic for veterinarians on their first day in practice with animals kept for scientific purposes as being *extremely important* (1) or only slightly less so (2), whereas, for male students, this score pattern was 29%, 48%, and 39%, respectively. With the exception of the scores of fewer than three female students, female and male students at all stages scored this topic as 6 or lower. The most dramatic difference in the score patterns was between male and female early students, with approximately 33% of female but only 7% of male students scoring this topic as *extremely important* (1).

### 3.6. Conscientious Objections

The interaction between gender and stage of study was significant (*p* = 0.030). The data show approximately 47%, 34%, and 33% of female early, mid, and senior students, respectively, scored an understanding of this topic for veterinarians on their first day in practice with animals kept for scientific purposes as *extremely important* (1) or only slightly less so (2). For male students, this score pattern was 12%, 33%, and 31%, respectively, across stages. Thus, there was a shift in male students to assign more importance to this issue as they progressed in their studies.

Furthermore, the least important score for this topic was 6 for females (with the exception of 1.1% of female students), and 8 for males (at the senior stage). The most dramatic difference in the score patterns was between male and female early students, with approximately 20% of female but only 2% of male students prioritizing this topic as *extremely important* (1).

Furthermore, no significant interaction was found for the interaction between gender and stage of study for the three variates: *AEC*, *the 3Rs*, or “*What Is a Research Animal?*”

## 4. Discussion

The survey results indicate veterinary students in Australia and New Zealand generally recognize the importance of Day One Competences for animals kept for scientific purposes in the key AWE topics identified. The AWE topics that were ranked the most important (*AEC Procedures or Requirements*, *the 3Rs*, and *Humane Endpoints*) may reflect a sound understanding by students of the importance of these issues given the relatively public debate about the use of animals for scientific purposes. Australian and New Zealand veterinary students are also exposed to the ethics of animal use for their own veterinary training (and the need to comply with the 3Rs), as well as to the associated regulatory framework (involving AECs).

Veterinary students in the current sample appear to acknowledge that their responsibilities extend to the wider social context of animals used by society and are not restricted to clinical competence. Given that a minority of veterinarians are likely to work as laboratory animal veterinarians, it seems that students appreciate the greater need to be knowledgeable in this area for times when their opinion is required, and as part of broader fields of veterinary work, including teaching, clinical research, and regulatory or compliance roles. We reported elsewhere that 9.7% of the students participating in this study thought they might work with research animals after graduation [23], and Kedrowicz et al. [24] reported a study in which 33% of veterinary students enrolled in a veterinary career course expressed “a great deal of interest” in a career in research and teaching in an academic setting, compared to 37% interested in specialist private practice. 

Veterinarians who are willing to provide input and advice on the management of research animals should be familiar with concepts such as cost-benefit analysis, experimental design, and application of the 3Rs [5]. The AWE issues associated with humane endpoints are also well recognized, and students’ understanding may stem from their appreciation of animal sentience and experience with similar issues in companion animal practice, where veterinarians must take some responsibility for appropriate end-of-life decisions. 

Responses showed that female students rated three topics—*Conscientious Objections*, *Humane Endpoints*, and *Euthanasia*—as significantly higher in importance for their initial competences than male students did across all stages of study. The most notable difference was that male early students were more likely to rate these topics as less important than female early students were: for *Conscientious Objections*, approximately 22% of female but only 2% of male students prioritized this topic as *extremely important* (1); for *Humane Endpoints,* the figure was 42% compared to 20%; and for *Euthanasia*, 33% compared to 7%. Moreover, the scores for these three variates showed a trend for the male scores for *extremely important* (1) to increase through the course, but for the female scores to decline slightly or stay relatively stable. However, there is caution in this interpretation, given the small sample size of males. For both males and females, combined and individually, more students prioritized the importance of Day One Competences (as assessed by a score of 1, or 1 or 2) for *Humane Endpoints* than they did for *Euthanasia*, and, in turn, *Conscientious Objections*. This may reflect students’ rationale that once they graduate, the need to conscientiously object would likely decline. 

When interpreting these findings, it should be remembered that this study investigated the importance that veterinary students place on an understanding of animals kept for scientific purposes as a Day One Competence, as opposed to how they might feel about the topics generally, or how they feel about the subject per se (e.g., being for or against animals being used for scientific purposes). Arguably, however, placing a greater importance on understanding a given topic most likely reflects the students’ views of the subject in general, and whether the students envisage an initial competence in this area to be relevant to their career. 

Previous studies have shown that females, including veterinary students, veterinarians, and scientists, show greater empathy, including by caring for animals and scoring pain higher in animals, than males in these categories [19,25,26,27,28,29,30,31,32]. The current findings supported such notions, in that female students in Australia and New Zealand were found to place more importance on understanding *Conscientious Objections*, *Humane Endpoints*, and *Euthanasia* as Day One Competences than their fellow male students did. Male students scored these AWE topics lower in the early stages of their veterinary courses than female students did, but their scores picked up over time. This may suggest that male students started from a lower empathy point in general, responded to AWE teaching material differently to females, or took longer to recognize the importance of these topics as Day One Competences. Nonetheless, it is interesting to note that, although female scores were higher overall for these topics, the males’ scores approached those of the female students over time. However, the current study took a cross-sectional look at students’ attitudes, so, without conducting a follow-up study on the same students, we are unable to confirm that such attitudinal changes occur over time.

This change in male scores might also reflect exposure to veterinary courses, teaching staff, and academics at the various universities that directly or indirectly reveal that these are important AWE issues to understand on day one. This hypothesis is supported by two recent Australian studies that showed the attitudes or empathy of veterinary students can be influenced with appropriate educational interventions [18,33]. However, it appears that educational interventions can also work the other way. A Swedish study by Hagelin et al. [34] demonstrated that a short course in laboratory animal medicine modified the views of half the students, with more than 26% becoming more supportive of animal use in research. Arguably, this attitudinal change may reflect a decrease in empathy, or show an increased understanding of and support for the regulatory safeguards in place, and it may tell us something about the general power of curriculum content in affecting student attitudes.

Several studies have suggested that veterinary students’ empathy for animals declines during the years spent at university, but the factors responsible for this are not well understood [18,23,25,26,35]. It may be that this loss of empathy reflects a greater exposure to some of the harsh commercial realities in veterinary medicine that may become apparent to students in their clinical years, especially in production animal medicine. A number of studies from the United States broadly support this idea [26,36,37]. In the current study, we did not see a decline in understanding of the importance of Day One Competences for the six AWE topics that were assessed, as they were ranked as relatively highly important by all students (both male and female), but whether this relates to empathy or a “need to know” requires further investigation.

Our analysis revealed no significant difference in the way male and female students rated three of the variates (*the 3Rs, AEC Procedures or Requirements*, and *“What Is a Research Animal?”*) across stages of study. For *the 3Rs*, approximately 61% of all students scored 1 or 2; for *AEC Procedures or Requirements*, approximately 69% of all students scored 1 or 2; and for *“What Is a Research Animal?”*, 42% of all students scored 1 or 2. Possible reasons for these findings include the perception by students that *AEC Procedures or Requirements* and the *3Rs* were less directly linked with “welfare” and were more technical or administrative in nature, or they lacked an understanding of the concepts. Further, the topic *“What Is a Research Animal?”* may have been confusing for students. The intent of the question was to investigate whether students thought understanding AWE issues associated with which species and types of animals are used for scientific purposes was important as a Day One Competence. The lack of gender difference to *“What Is a Research Animal?”* in our study may be supported by an earlier Australian study by Phillips and McCulloch [19] that found there were no gender differences in their attribution of sentience to the different animal species. 

Although student participation in the current study was encouraged, the students who did respond to the questions regarding animals kept for scientific purposes that have been discussed within may have been particularly committed to animal welfare or feel strongly against the use of animals in science and are hence more likely to complete those questions, as they saw the survey as an opportunity to voice their strong opinions on the matter. It is also worth noting that females are generally more likely than males to respond to survey invitations [18]. That said, the gender balance of 79% females in the current sample appears consistent with the gender composition of new graduates in Australia, which is around 80% female [18]. Although opinions were captured using a Likert-scale response, inviting some additional qualitative answers may have allowed us to better understand the reasons for certain topics being scored lower or higher.

This study was intended to be the start of a review of Day One Competences for veterinary graduates in this important, and often contentious, area of animals used for scientific purposes. While it is widely acknowledged that veterinarians have an integral role to play in ensuring the use of animals for scientific purposes is humane, ethical, and complies with relevant legislation, it is not yet established how best to achieve Day One Competences effectively and uniformly throughout veterinary schools, nor whether veterinary students fully understand the importance of their roles in this area. 

As veterinary educators, our view is that the welfare of animals used for scientific purposes is best safeguarded by veterinary involvement in evaluating proposed experiments on animals and in providing direct supervision over experiments [38]. While the latter is not a legal requirement in Australia or New Zealand, more involvement by veterinarians should be promoted in the oversight of animal care, and as AEC members. This has the potential to improve the welfare of animals, and to provide fulfilling career paths for veterinarians. 

In conclusion, the findings of the current survey suggest that veterinary students are suitably aware of the ethical challenges and obligations surrounding the use of animals in research and education. Given that only a small proportion of students might be expected to eventually work in the field, the remainder of the student body appears well placed to act as professional experts and advocates within the community amidst discussions and developments in society on the use of animals for scientific purposes.

## Figures and Tables

**Table 1:** The institution and number of veterinary students who responded to the survey.

Institution	Number of Students (Total, Percentage)
The University of Sydney	147 (600, 24.5%)
Massey University	141 (500, 28.2%)
James Cook University	91 (350, 26%)
Charles Sturt University	84 (295, 28.4%)
The University of Queensland	68 (609, 11.1%)
The University of Adelaide	119 (317, 37.5%)
The University of Melbourne	52 * (259, 20%)
Murdoch University	116 (390, 29.7%)

* Only first and second year students surveyed.

**Table 2:** The ranked importance assigned to the animal welfare and ethics (AWE) topics and the log-linear regression analysis related to animals kept for scientific purposes by veterinary students.

AWE Topic	Overall Ranking	Percentage of Students Scoring Topic 1 or 2	Log Linear Regression Analysis for Each Variate in Survey Responses as a *p* Value		
			Stage Gender Score	Stage Score	Gender Score
AEC (Animal Ethics Committee) Procedures or Requirements	1	68.6	0.571	0.058	0.247
3Rs (Replacement, Refinement and Reduction)	2	61.3	0.991	0.467	0.171
Humane Endpoints	3	60.6	0.026	ND	ND
Euthanasia	4	48.5	0.014	ND	ND
What Is a Research Animal?	5	41.8	0.093	0.989	0.061
Conscientious Objections	6	36.9	0.030	ND	ND

ND: not determined.
